# Psychological Counseling among University Students Worldwide: A Systematic Review

**DOI:** 10.3390/ejihpe13090133

**Published:** 2023-09-14

**Authors:** Silvia Cerolini, Andrea Zagaria, Costanza Franchini, Vito Giuseppe Maniaci, Alexandro Fortunato, Chiara Petrocchi, Anna Maria Speranza, Caterina Lombardo

**Affiliations:** 1 Department of Psychology, Sapienza University of Rome, 00185 Rome, Italy; andrea.zagaria@uniroma1.it (A.Z.); caterina.lombardo@uniroma1.it (C.L.); 2 Department of Dynamic and Clinical Psychology, and Health Studies, Sapienza University of Rome, 00185 Rome, Italy; costanza.franchini@uniroma1.it (C.F.); maniaci.1900359@studenti.uniroma1.it (V.G.M.); alexandro.fortunato@uniroma1.it (A.F.); annamaria.speranza@uniroma1.it (A.M.S.); 3 Department of Developmental and Social Psychology, Sapienza University of Rome, 00185 Rome, Italy; chiara.petrocchi@uniroma1.it

**Keywords:** psychological counseling, university students, psychological intervention, university counseling services, systematic review

## Abstract

University counseling services (UCSs) are actively involved in mental health assessment and in supplying interventions aimed at preventing, facing and possibly overcoming psychological problems. However, we do not have a global overview of psychological counseling among universities. This systematic review aims at reviewing the literature on university psychological counseling, including articles documenting: (1) mental health and attitudes regarding help-seeking behaviors and UCSs among university students or counselors, (2) the description of protocols/services among UCSs, (3) the efficacy of psychological counseling/interventions among university students (both face-to-face and internet-delivered interventions). The study followed PRISMA guidelines and was registered on PROSPERO. After defining inclusion and exclusion criteria, a literature search was conducted, identifying 7085 records. Finally, 152 articles met the review eligibility criteria and were included in the qualitative synthesis. Results are divided into seven thematic topics that emerged during the analysis of the literature. The results mainly showed that face-to-face and web-based counseling/psychological interventions improve university students’ mental health. Cross-sectional studies showed that many biases exist toward help-seeking behaviors, especially among international students. Both students and counselors must strive to overcome cultural barriers. Available resources for UCSs are scarce and need to be strengthened, as well as efficacy studies through randomized clinical trials.

## 1. Introduction

Emerging adulthood can be a challenging and distressing life stage for many young adults [1,2]. In high-income countries, this coincides with attendance in university studies [1,3]. During this developmentally crucial period, an age peak for the onset of mental disorders may be observed [4]. According to the WHO’s World Mental Health International College Student (WMH-ICS) initiative, around 35% of first-year university students screen positive for at least one lifetime mental disorder, while 31% screen positive for at least one 12-month mental disorder [5]. In addition, meta-analytic evidence signals that about 22.3% of university students report suicidal ideation [6]. Most frequently, students experience subclinical psychopathological symptoms and psychological problems. For example, undergraduate students often report poor sleep and/or insomnia [7], which has been associated with stress [8]. Similarly, students report experiencing low self-esteem, test anxiety, procrastination, perfectionism, and stress, which have been associated with mental disorders [9]. Psychosomatic symptoms (i.e., headaches, stomachaches, etc.) are frequent complaints in association with increased internet use [10]. Moreover, university students tend to engage in more risky behaviors than healthy behaviors [11,12]. All of these factors mentioned above may negatively affect academic performance and predict a higher likelihood of college dropout [13,14].

Although several barriers to treatment have been identified so far, such as treatment stigma and attitudes toward mental health help-seeking [15], university students are increasingly engaging in help-seeking behaviors and accessing university counseling services (UCSs), often overwhelming these services [16]. The heavy demand on UCSs coupled with limited funding and lack of staff may result in long waiting lists in which students wait up to several weeks or months before receiving support, exacerbating the risk of adverse psychological and physiological responses [17]. For this reason, to make UCSs more accessible for all students, many studies have explored the effectiveness of internet-delivered psychological interventions, which could potentially overcome these barriers. With available technologies (i.e., websites, apps, videoconferencing, etc.), such interventions may offer several advantages, including ease of access, low costs, anonymity, no waiting list and easily monitored progress [18], that may safeguard these services and the health of students approaching them. 

Moreover, the global COVID-19 pandemic has worsened the mental health of university students [19], with more than 50% experiencing clinically significant levels of psychological symptoms such as anxiety and depression [20]. As a result, there is an urgent need for increased investments in and reinforcements of timely mental health care [21], which many UCSs are carrying out in different countries [22]. It is crucial for UCSs to continuously evaluate students’ mental health and well-being, identify and remove any barriers to treatment (e.g., economic or cultural obstacles, prejudices, and stereotypes), and provide tailored psychological interventions to target their specific needs. 

Despite its growing importance, however, there is currently no comprehensive global overview of psychological counseling among universities. To the best of our knowledge, only a few systematic review studies analyzed specific aspects of this macro-topic, focusing on digital mental health interventions [23], the effectiveness of mobile app-based psychological interventions [24] and the use of mental health services [25] among university students. The first review included 88 articles and found that digital mental health interventions can be effective for improving depression, anxiety, and psychological well-being among college students [23]. The second review included only 19 articles and showed that mobile apps for mental health intervention exist and demonstrate good acceptability and feasibility, demonstrating efficacy among students [24]. The third review synthetized, both qualitatively and quantitatively, evidence from 44 studies analyzing the proportion of university students using mental health services, and how this varies by service type [25]. They found a large variety of available services used by varying proportions of students. However, the included studies were mostly conducted in the USA, limiting the generalizability of their results [25]. 

Until now, no study has tried to give an overview of the situation of counseling worldwide. This review was designed and conducted to that end. Specifically, we aimed to review articles addressing: (a) attitudes regarding help-seeking behaviors or counseling services among university students or counselors, (b) mental health among university students who are accessing or attending counseling services, (c) the effectiveness of psychological counseling/interventions among university students, and (d) the description of protocols/good practices/purposes or counseling services among student communities and universities. Through an examination of the geographical distribution, actuality, and typology of contributions, as well as the methodological quality and robustness of studies currently published in the existing literature, this review aims to provide insight into the functioning of UCSs, identify gaps and limitations, and highlight areas for future research. Namely, this review can shed light on the attitudes, barriers, and stereotypes concerning health-seeking behaviors, the mental health of university students who utilize UCSs, and the effectiveness of psychological interventions in this population. By outlining the protocols, good practices, and purposes of UCSs, this review can also help identify strategies to enhance the support offered to university students, particularly in light of the ongoing COVID-19 pandemic, which has had a well-established, profound impact on their mental health. 

## 2. Materials and Methods

### 2.1. The Present Review

This systematic review offers a brief overview of psychological counseling among university students worldwide. The study followed the Preferred Reporting Items for Systematic Reviews and Meta-Analyses (PRISMA) guidelines [26]. It was registered on the International prospective register of systematic reviews (PROSPERO registration ID: (masked for review)).

### 2.2. Search Strategy

A systematic literature search of published studies was performed from inception up to 20 September 2022, using the following electronic databases: Medline, PubMed, CINAHL, PsycINFO, and Scopus. The search string was: ((psycholog* counsel* OR psycholog* intervention OR university counsel* service* OR university counsel* center* OR university counsel* centre*) AND (university student* OR college student*)). The detailed search strategy is reported in Supplementary Document S3. The literature search was performed by the first author (SC). After removing duplicate records using an automatic tool (https://rayyan.ai, accessed on 28 August 2023), two authors (CF and VM) independently screened the titles and abstracts in the first round (from October to November 2022). In the second round of screening, two authors (SC and AZ) independently screened the full-text articles for eligibility against the pre-defined inclusion and exclusion criteria (from November to December 2022). Cohen’s k value was calculated to evaluate interrater reliability (i.e., a value above 0.80 shows a substantial agreement, [27]). The two judges (SC and AZ) discussed inconsistency in the appraisals and resolved disagreements by discussion. Finally, the full-text articles that met the inclusion criteria were included in the systematic review after reaching a total agreement between the reviewers (December 2022).

### 2.3. Inclusion and Exclusion Criteria

The inclusion criteria were defined as follows: (a) studies published in the English language; (b) studies involving or related to university students aged 18 years or older or to the university student community (including health care providers); (c) studies focused on brief psychological counseling/intervention (i.e., a determined or undetermined number of sessions that should not be short-term or standardized psychotherapy and not be targeted at specific mental health problems or diagnoses). To clarify the eligibility of the studies, the following exclusion criteria were applied: (a) studies addressing counseling or brief intervention exclusively related to a specific topic or a specific clinical population (e.g., alcohol, drugs, other substances, sex or sexual abuse, suicide, covid, autism, PTSD, etc.); (b) studies addressing psychotherapy; (c) studies related exclusively to the COVID-19 pandemic; and (d) prevalence studies or cross-sectional studies addressing only mental or physical health of university students without any reference to counseling services. All types of study (e.g., randomized controlled trial, cross-sectional, qualitative study, text and opinion, etc.) were considered eligible. No time or publication date restriction were applied. Grey literature (e.g., conference abstracts, unpublished reports, non-peer-reviewed articles), systematic reviews and editorials were excluded. Lastly, studies conducted in healthy and clinical samples were considered eligible unless counseling only focuses on a specific diagnosis. Eligibility criteria are synthesized in Table 1.

### 2.4. Quality Assessment

To assess the trustworthiness, relevance and results of the articles included, the Joanna Briggs Institute’s critical appraisal tools were used. Since all study designs were considered eligible, their methodological quality was assessed using the Critical Appraisal checklists for (a) Analytical Cross-sectional Study; (b) Cohort Studies; (c) Randomized Control Trials (RCTs); (d) Qualitative Research/Prevalence Studies; (e) Quasi-experimental Studies; (f) Text and Opinion; (g) Case reports (see https://jbi.global/critical-appraisal-tools, accessed on 12 January 2024). Each tool had a different number of items, ranging from 6 (for text and opinion) to 13 (for RCT), for assessing the methodological quality of the included papers. Item content varied according to the study design and assessed specific quality facets, such as the validity and reliability of the measurement, strategies for handling missing data, strategies to deal with confounding variables, the completeness of information on the setting and characteristics of the sample, the clarity of the inclusion and exclusion criteria, and whether the opinions expressed were supported by scientific evidence (for text and opinion papers). Items were coded with three possible response options: “Yes”, “No”, or “Unclear”. The first (SC) and the second (AZ) authors independently performed the quality appraisal, and disagreements were resolved by discussion (January 2023).

### 2.5. Data Extraction

All relevant details for each selected study were extracted using a standardized datasheet form (from January to February 2023). The following information was extracted: authors, year of publication, topic, sample size, mean age, gender distribution, ethnicity, university status (freshman, sophomore, doctoral student, etc.), field of study, marital status, length of intervention, type of intervention, type of counselor, outcome, and study location. Each piece of information was reported as mentioned in the text or coded as “unclear” or “not available”. Moreover, after carefully screening the literature collected thus far, the “Topic” section was categorized according to the following coding system: 1. Mental health, help-seeking behaviors and attitudes among university students; 2. Description of counseling services, good practices and purposes to improve the services; 3. Effectiveness of counseling and psychological interventions; 4. Internet (web-based) delivered intervention; 5. International students; 6. Counselors and health care providers; 7. Other. Similarly, the “outcome” section was coded as follows: (1) mental health and well-being, (2) attitudes toward help-seeking behaviors or counseling, (3) psychopathological symptoms and mental distress indicators, (4) academic achievement and satisfaction, (5) other. A cross-check of the extracted information was performed by a second reviewer.

## 3. Results

### 3.1. Literature Search

**Figure 1:**
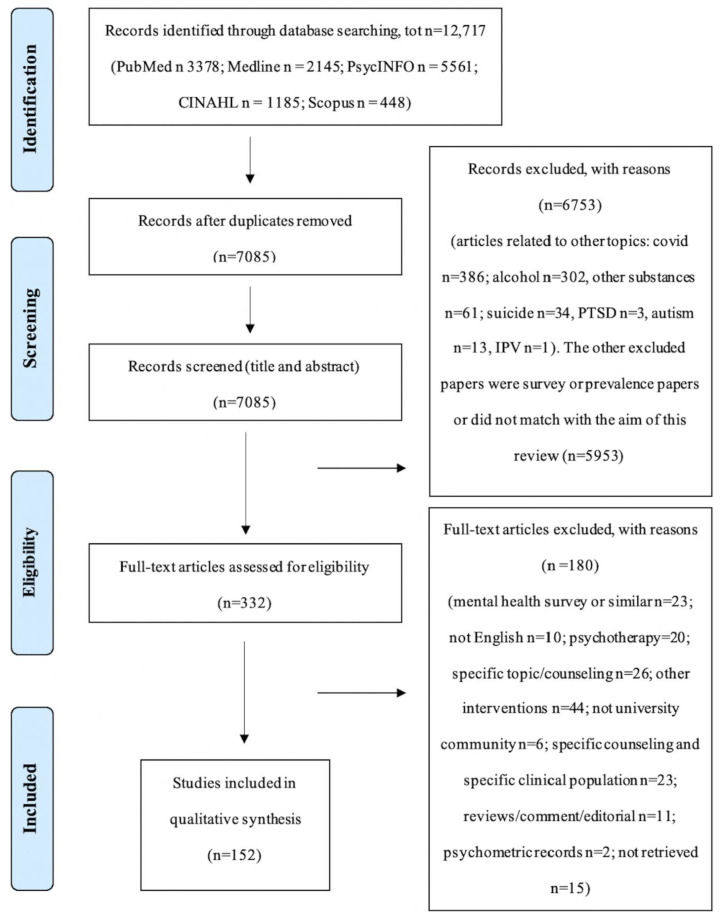
PRISMA flow diagram.

The literature search yielded 12,717 records from the selected electronic databases, which decreased to 7085 after removing duplicates. The identified records were screened for relevance using titles and abstracts. A total of 332 records passed the first screening phase, and their full texts were assessed for eligibility. The average percentage of articles that contained “unclear” or “not available” data was 4.5%. Finally, 152 studies met the review eligibility criteria and were included in the narrative synthesis (the complete references list of the included articles is available as Supplementary Document S1). In this last full-text screening phase, a total agreement between the two reviewers appeared (i.e., Cohen’s k = 1). The procedure is illustrated through the PRISMA flow diagram alongside reasons for exclusion and numerical reports (Figure 1).

### 3.2. Study Characteristics

**Figure 2:**
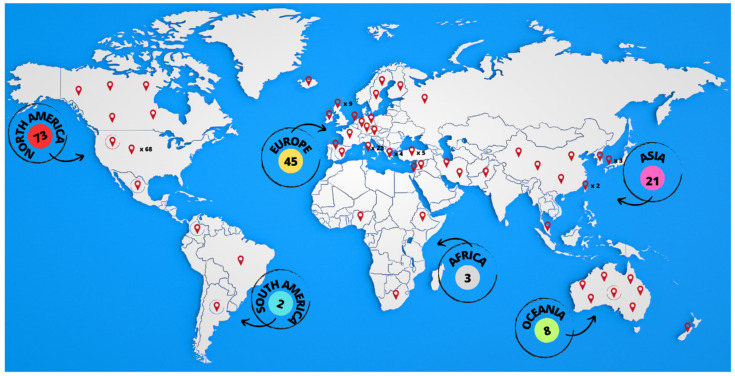
The image shows all of the countries in which studies had been conducted. Among them, four multination studies were counted individually in the total of studies for each country, although the icons show all of the places where the studies were designed and/or conducted (e.g., one multination study conducted in Spain and Germany was considered as one study in the total number of studies in “Europe”, but the icons were represented over both Spain and Germany).

Among the 152 included articles (references are provided in Supplementary Document S1), data extraction documented the presence of eight types of study design as follows: cross-sectional studies (N = 50), longitudinal studies (N = 3), RCTs (N = 27), quasi-experimental studies (N = 34), text and opinion (N = 19), prevalence studies (N = 10), qualitative studies (N = 7), and case reports (N = 2). Concerning the geographic distribution of contributions, most of them were conducted in North America, followed by Europe, Asia, Oceania, Africa and South America. Few studies were conducted, or projects launched, across multiple countries [28,29,30,31]. Figure 2 illustrates the geographical distribution of the included studies.

Among all of the studies, including participants from the university community and providing a sample size, a total of 338,644 university students and a total of 2145 counselors were enrolled (see Table 2 for detailed sample sizes for each type of study). Table 2 summarizes the details regarding the types, number and sample sizes of the studies included.

University students’ mean age was 23.61 years, and the percentage of females was 64.83%. The most prevalent racial/ethnic group was Caucasian, but most of the studies included several ethnical minorities, especially those addressing topic 5, “International students”. Most of the students were undergraduates or first-year students. Even if in the minority, students at other academic levels were also included (e.g., doctoral students, graduate and post-graduate students). Their fields of study were heterogenous, and marital status was generally not reported. 

Demographic information about counselors has been underreported. However, a large study documented that counselors were mostly female, in their early thirties, Caucasian, and had a Ph.D. or a Psy.D. Most of them were licensed clinicians and members of UCS staff, though some were interns, Ph.D. or practicum students [32].

As regards the length of interventions, the RCTs documented that, on average, the duration was 5.96 sessions/modules, often held weekly. Also, quasi-experimental studies confirmed this estimated average length (M = 5.81 sessions/modules). The most prevalent outcome measured by all of the studies examined were mental health and well-being, followed by psychopathological symptoms and mental distress indicators, attitudes toward help-seeking behaviors or counseling, and academic achievement and satisfaction. About half of the selected studies addressed multiple outcomes (50.66%). A detailed table presenting all of the extracted data is available in Supplementary Material Document S2.

### 3.3. Quality Assessment Results

The quality assessment results are summarized in Supplementary Document S2, revealing a highly heterogeneous picture of the included studies. In cross-sectional studies, standardized and reliable exposure and outcome measures were the main strengths. However, areas for improvement were identified in the lack of consideration of potential confounders and inadequate strategies to account for them, e.g., by including them as covariates in hierarchical regression analyses. For RCTs, the main strengths were the proper implementation of the randomization procedure and reliable and valid measurement of outcomes in both groups. Nonetheless, weaknesses were associated with the absence of blinding among treatment deliverers and outcome assessors, which could introduce bias and threaten the study’s internal validity. Most of the quasi-experimental studies had valid and reliable outcome measures, consistent measures of the outcomes across different groups, and a clear conceptualization of predictors and outcomes. However, weaknesses included incomplete follow-up and inadequate strategies for handling missing data.

Regarding text and opinion papers, most studies clearly identified the source of the opinion and were conducted by individuals with adequate expertise in the field. The analytical logic underlying the opinion was deducible and supported by scientific literature. Prevalence studies exhibited strengths such as appropriate sampling frames to address the target population and sufficient data analysis coverage of the identified sample. However, weaknesses included unreliable measurement of the condition of interest. Concerning qualitative studies, one of the observed weaknesses was the lack of consideration given to the potential bidirectional influence between the researcher and the research in the evaluation of the outcomes, which could introduce a bias and compromise the validity of the findings. Concerning case reports, crucial information such as patient’s demographic characteristics, clinical history, diagnostic tests, and intervention procedures were not clearly described, which hindered the possibility of drawing accurate conclusions and underscored the need for a cautious interpretation of the findings. Finally, cohort studies exhibited strengths such as enrolling participants free of the outcome at the study’s onset and employing reliable and valid outcome assessments. However, the main weakness identified was that the two groups were dissimilar and/or recruited from different populations. Moreover, exposure measures were not clearly defined and described in detail.

### 3.4. Synthesis of the Content of the Included Studies

Table 3 shows the seven thematic topics that emerged during the systematic analysis of the literature. These are useful in summarizing the contents of the articles included and facilitating their presentation. 

#### 3.4.1. Topic 1: Mental Health, Help-Seeking Behaviors and Attitudes toward Counseling among University Students

Forty-nine studies exclusively addressed topic 1, “Mental health, help-seeking behaviors and attitudes among university students”, while 17 studies addressed topic 1 in conjunction with other issues, such as topic 2 “Description of counseling services, good practices and purposes to improve the services” (N = 2), topic 3 “Effectiveness of counseling and psychological interventions” (N = 4), topic 4 “Internet (web-based) delivered intervention” (N = 4), topic 5 “International students” (N = 3), and topic 6 “Counselors and health care providers” (N = 3). One study addressed multiple topics (i.e., topics 1, 2 and 6, N = 1).

The subjects were mainly addressed within analytical cross-sectional studies (N = 44), followed by prevalence studies (N = 7), quasi-experimental studies (N = 5), qualitative studies (N = 5), cohort studies (*n* = 2), RCT (N = 1), and case reports (N = 2). The main body of studies had been conducted in North America, principally in the USA, but all of the countries contributed to this topic.

As mentioned in the inclusion and exclusion criteria, articles addressing exclusively mental health among university students were excluded due to the enormous number of papers involved and the lack of focus on counseling, the main topic of this systematic review. Consequently, the retained articles included measures of mental health and psychological symptoms but mainly focused on attitudes and concerns toward counseling, counselors, help provider sources, and help-seeking behaviors [33,34,35,36,37], expected activities and perceived needs [38], barriers and demographic predictors to receive professional mental health care [39]. Demographic characteristics of students and counselors such as gender or ethnicity, as well as acculturation, confidence and openness to mental health and counseling, and family history of mental illness constituted the principal barriers to help-seeking behaviors [33,34,35,36,37]. A study including 1135 undergraduate university students from Ethiopia [39] found that the top five barriers to receiving professional mental health care among students were (a) thinking the problem would get better by itself, (b) being unsure where to go to get professional help, (c) wanting to solve the problem on their own, (d) denying a mental health problem, and (e) preferring to get alternative forms of mental care. 

Some studies investigated the mental health [40] and coping strategies [41] of help-seeking students and the differences between those who had accessed UCSs versus those who had not [42,43], showing more maladaptive psychological functioning in the first group [44]. Moreover, among help-seeking students at UCSs, demographic factors such as being first-generation students were associated with more academic distress, work hours, and financial distress than being non-first-generation students [45]. Some other authors explored the attitudes, stigma, and intentions toward seeking online and face-to-face counseling [15], suggesting that face-to-face counseling is, overall, considered a more favorable method of service delivery compared to online counseling and that males reported significantly lower value (lack of utility, benefits, and usefulness of services) and significantly higher self-stigma toward both forms of counseling in comparison to females. Some studies measured the frequency of access at UCSs and mental health services [46] and variables related to UCS use [47]. One study, including a large sample size, showed that symptoms of anxiety, depression and alcohol use disorder were the main predictors of UCS use, followed by stressful life events [48]. Among the factors associated with help-seeking behaviors and UCS access, the characteristics of counselors were investigated (e.g., demographic characteristics like gender, age and ethnicity) [28,34]. Some authors [49] identified the dimensions of expertness, attractiveness, and trustworthiness underlying students’ perceptions of the counselor. Qualitative studies [50] explored which measures and interventions (also referring to the existing literature) university students found helpful and acceptable themselves, thus possibly contributing to increasing user acceptance and utilization and the effectiveness of these services. In this study, for example, participants approved the interventions designed to improve mental well-being and highlighted the need for a broader psychological support system, longer opening hours at UCSs, possibly compatible with their schedules, and more information regarding mental health problems and interventions. Another qualitative study [36] focused on barriers to help-seeking behaviors, identifying the following as the significant barriers: (1) misconceptions and distrust of on-campus counseling (lack of trust in counselors), (2) stigma toward mental illness, (3) low mental health literacy, and (4) inaccessibility of mental health services, which were consistent with most of the previously mentioned studies.

#### 3.4.2. Topic 2: Description of Counseling Services, Good Practices and Purposes to Improve the Services

Eighteen studies exclusively addressed topic 2, “Description of counseling services, good practices and purposes to improve the services”, and six studies in conjunction with other issues, precisely with topic 1 (N = 2), topic 5 (N = 1), topic 6 (N = 1), topic 1 and 6 (N = 1). Mainly, this argument was addressed through text and opinion design (N = 18), with most studies having been published in North America or Europe.

Some articles aimed at presenting UCSs in a specific geographical area [51], emphasizing the activities offered (e.g., individual and group psychological support, crisis intervention, research, prevention, the support provided to students with special needs, [52], and calling for more descriptive investigations of the diverse available counseling programs and more outcome research to assess the effectiveness of these services [53].

Some authors were concerned with describing how psychological orientations/approaches can support university counseling [54,55], especially brief psychological interventions that imply a very limited number of sessions [56], also presenting examples of counseled cases [57,58]. Others focused on the challenges and opportunities of UCSs for students in specific conditions. For instance, students with psychological issues before and during their study-away experiences [59], during summer schools [60], or when preparing for final exams [61] could be welcomed and helped by ad hoc counseling services and even innovative programs (e.g., a therapy dog program for an outreach activity to reduce stress as students prepare for final exams, as described by [61]). Still, others stressed the need to expand the expertise of counselors and improve training on specific issues, to provide more comprehensive services (e.g., training in neuropsychology, as proposed by [62]; multidisciplinary and integrative approaches, as proposed by [63]; or skills in “psychosociocultural” diversity, as proposed by [64]). 

Finally, UCSs may be used to build a community of research and practice. This is the case of “The Center for Collegiate Mental Health” [65], which aims at creating a collaborative, long-term, multidisciplinary network between UCSs and personnel involved to (a) describe student mental health at a national level, (b) conduct large-scale clinical research, and (c) improve the range of clinical tools available to practitioners in the higher-education setting. Ref. [65] presented a model of a practice-research network that may enhance our ability to understand and improve UCSs and foster further collaborations between clinicians and researchers.

#### 3.4.3. Topic 3: Effectiveness of Counseling and Psychological Interventions

Fifty-three studies tested the effectiveness of counseling and psychological interventions. However, among them, 14 studies tested the effectiveness of web-based interventions (topic 4), which will be the focus of the next paragraph. Three studies addressed topic 3 in conjunction with topic 1, and the other three studies in conjunction with topic 6. Only one study addressed topic 3 together with topic 5.

Quasi-experimental studies (N = 30) and RCTs (N = 18) were the most-used study designs. One study used a qualitative method, three studies used cross-sectional methods, and one study a longitudinal method. North America and Europe are the countries where most of the studies have been conducted on this topic. 

A percentage of the selected articles specifically investigated the effects of individual face-to-face counseling, provided mainly by UCSs, on students’ mental health and/or academic performance [66,67,68,69,70,71,72], often using retrospective case studies [70,73,74,75,76,77]. However, only in a few cases, the effectiveness of group counseling was also tested (e.g., [78,79,80]). Different typologies of counseling were documented: psychodynamic-oriented counseling (e.g., [68,81]), cognitive behavioral oriented counseling (e.g., [78,79,80,81,82]) or multiple orientations counseling (e.g., in the case of data gathered by the Center for Collegiate Mental Health, [76,77]). In all of these cases, for both individual and group counseling, there was strong evidence of their effectiveness in improving students’ mental health or academic achievements. Nevertheless, some empirical evidence also indicated that there were exceptions; for example, counseling students with disabilities may have had some limitations since they demonstrated significantly fewer reductions in levels of psychological and academic distress than their nondisabled peers throughout treatment [77].

Despite some limitations in counseling intervention, similar types of brief psychological interventions were also proven to be robust and beneficial in improving students’ well-being/academic performance and reducing psychopathological symptoms (e.g., [83,84,85,86]), or in helping them with some aspects of their life associated with potential risks (e.g., one study [87] showed the effectiveness of a preventive counseling program for the positive use of social network sites and psychological hardiness among students at risk). Also, in this case, some of these short psychological interventions were based on specific therapeutic approaches and techniques, such as cognitive behavioral therapy (CBT), both in individual [82] and group formats [88], and acceptance and commitment therapy (ACT, [85]). Finally, some studies that tested the effectiveness of counseling-related interventions could be distinguished from the chorus. For instance, one study [89] tested the therapeutic effects of sharing Minnesota Multiphasic Personality Inventory-2 (MMPI-2) assessment results with clients, evidencing its potential beneficial impact on self-esteem and symptomatic distress. Others documented the effectiveness of the counseling intervention in facilitating personal growth [90] or focusing on self-understanding and interpersonal interactions to prevent psychosocial distress [91]. More recently, some authors showed that positive rumination group training was helpful in improving mental health and reducing attentional bias toward sad faces [92], and that a positive psychology group intervention program could be effective in helping students experience positive emotions [93].

#### 3.4.4. Topic 4: Internet (Web-Based) Delivered Intervention

Twenty-six studies focused on web-based interventions, of which 10 were RCTs, four were quasi-experimental studies, four were analytical cross sectional studies, one was a qualitative study and seven presented new protocols/tools (see Topic 5 “Other”). These studies were mostly conducted in Europe, followed by North America, Oceania, Asia and South America.

All of the RCTs investigated the effectiveness of online psychological interventions declined in several ways: mostly programs based on online modules (e.g., [94,95,96,97,98]), websites interaction (e.g., [99]), use of mobile apps (e.g., [100]), and face-to-face meetings followed by online exercises and interaction (e.g., [101]). Only one quasi-experimental study without a control group tested the effectiveness of online counseling delivered by a physical counselor due to pandemic needs [22]. Except for the latter studies [22,101], all of the other programs did not require intervention delivery by a physical counselor and usually had a weekly schedule. Some of them were ACT-oriented (e.g., [97,98,101]) and CBT-based (e.g., [96,102]), offering a wide range of evidence-based techniques and options for the promotion of well-being and the transdiagnostic prevention of common mental disorders and psychological distress among university students (e.g., [94,95,96]). Automated web-based delivery and saving resources allowed the distribution of the interventions to vast and multi-national samples (e.g., [96,98,100]). However, the set nature of the modules and online interventions, in general, could limit the appropriate response to the personal needs of each student, whereby mixed interventions in which interaction with a physical counselor would be expected at different times of the program might be desirable (as in [101]).

#### 3.4.5. Topic 5: International Students

Only five studies focused on international students’ mental health, help-seeking behaviors, access to UCSs and interactions with counselors. All of the studies were conducted in North America or in Oceania. 

Specifically, two prevalence studies examined (1) the needs, concerns and life changes of international students [103] and (2) the use of UCSs in this population [104].

The first study, including 640 international students, showed that their needs and concerns could be high and common (especially those regarding immigration regulations and visa requirements) but also differentiated depending on the geographical origin (i.e., different educational concerns and experiences regarding personal changes and daily challenges). The second study, including 834 international students, documented an underutilization of UCSs by this minority, suggesting mistrust or stigma toward help-seeking behaviors, and lack of knowledge or culturally appropriate services at UCSs. This probably contributes to an increase in the severity of initial psychopathological symptoms upon UCS access, less engagement in counseling sessions and the maintenance of clinically significant distress in certain areas (e.g., social role functioning) after treatment (as reported by retrospective results collected from 5472 students and 66 counselors [105]). These results also agreed with previous findings [106] on 1166 ethnic minority college students. They experienced the greatest distress, with less care and benefits from counseling than their Caucasian peers. Although it included a very small sample of only six international students, a recent pilot study [107] tried to overcome these barriers by promoting an 8-week strength-based support group called the International SuperHERO. This intervention promotes students’ well-being by increasing their hope, efficacy, resiliency, and optimism. Students’ feedback and qualitative results showed encouraging and promising results for this innovative type. Although few, these studies help to extend our knowledge about the reality of student diversification underpinning the majority of universities in the world.

#### 3.4.6. Topic 6: Counselors and Health Care Providers

Eleven studies focused on UCS counselors and health care providers, often in conjunction with topics 1, 2 and 3, mostly conducted in North America. Studies collected in this section included both counselors’ personal perceptions and experiences, or reports on clients’ mental health and counseling experiences. Starting from cross-sectional results, an extensive investigation [32] examined archived data collected via the Center for Collegiate Mental Health, involving 1308 counselors from 84 UCSs who rated the concerns presented by 53,194 clients. Their findings offer an extensive and generalizable description of university students’ concerns and reasons for seeking help at UCSs, as reported by clinicians’ evaluations. Anxiety, depression, stress, family, and academic performance were the most prevalent reasons for asking for help. Also, suicidality was described as an area of concern for 8.4% of students. Moreover, a qualitative study [108] analyzed antecedents and consequences of academic procrastination using the content of 12 semi-structured interviews administered to experienced university counselors. 

On the other side, focusing on counselors’ needs and staff’s perceptions of the UCSs, one study [109]) indicated that one of the strengths of UCS staff is to have a highly motivated, well-educated team working harmoniously. Conversely, the weaknesses of UCSs include a lack of personnel and organization, work overload and poor coordination between services. Developing cooperation and creating interdisciplinary committees or working for groups with clear definitions of roles and tasks among the staff may help strengthen UCSs. Furthermore, to deepen the expertise and attitudes of counselors, a mixed-method study [110] measured perceived grief counseling skills and comfort with grief counseling in 171 university counselors. Their findings highlighted the need for research, training, and clinical work on this theme, which is still scarce. In the same context, a quasi-experimental study [111] explored the effectiveness of a brief, empirically validated, clinician-administered suicide prevention intervention, namely the Safety Planning Intervention (SPI), for 12 university counseling centers’ direct service staff. Confidence in assessment and intervention skills and satisfaction and experience with, and implementation of, what they learned in their counseling practice were, therefore, measured, producing encouraging results for implementing and disseminating this practice to reduce suicide risk among college students.

#### 3.4.7. Topic 7: Other

The category “Other” was used for group studies presenting, for example, protocols, clinical practices and tools useful for university counseling or psychological interventions and included 10 articles, mostly presented by European universities. Among them, seven presented RCT protocols (e.g., [96,112,113], one introduced a new technology-based counseling environment (a virtual reality platform; [114]), and two presented psychological intervention program protocols.

## 4. Discussion and Conclusions

This systematic review aimed to explore the functioning of UCSs worldwide by reviewing the literature on attitudes and behaviors toward, and descriptions and effectiveness of, brief psychological counseling. The findings synthesized in the previous sections may enhance our knowledge of students’ mental health and help-seeking behaviors and highlight critical differences among members of this population. In fact, university students are going through a particular phase of life and simultaneously face various challenging experiences, such as exams, stressful internships, studying abroad, and gaining economic and life independence. Since the need for support expressed by university students is growing, UCSs are proving to be one of the most important sources of guidance and support, although several improvements need to be made.

Firstly, the results presented in the “Topic 1: Mental health, help seeking behaviors and attitudes toward counseling among university students “ section, consistent with previous findings (e.g., [3]), suggest that university students’ mental health requires attention from health professionals, given the increase in psychological symptoms and persistent barriers to help-seeking behaviors in this population. In fact, a good proportion of university students use UCSs, as confirmed also through recent meta-analytic findings by [25], but many barriers still exist and may prevent access. These barriers may be related to (1) misconceptions and distrust of on-campus counseling (lack of trust in counselors), (2) stigma toward mental illness, (3) low mental health literacy, and (4) lack of access to mental health services (e.g., [36]). 

Results presented in the “Topic 2: Description of counseling services, good practices and purposes to improve the services” section agreed in suggesting that UCSs must be reinforced and empowered in the areas of prevention, psychological support, crisis intervention and research (e.g., [52,53]). Due to the increased use of UCSs by the university population [25], there is an urgent need to expand the services offered and create collaboration and networks among UCSs. This is the case of The Center for Collegiate Mental Health [65], which aims at describing student mental health at a national level, conducting large-scale clinical research, and improving the clinical tools available. 

As highlighted by different studies included in the “Topic 3: Effectiveness of counseling and psychological interventions” and “Topic 5: International students” sections, many counseling interventions are proving effective in improving the mental health of university students both face-to-face and online. However, expanding and refining the services offered by UCSs (e.g., prevention programs, transdiagnostic intervention, multicultural training for counselors, etc.), especially those targeting students with specific needs or disabilities, including visible and non-visible disabilities [77], and international students [107], may be a first step toward managing the demand and improving outcomes, which are still scarce in these populations [77,105,106]. The scarce number of studies related to international students (*n* = 5) are in line with recent meta-analytic findings about students’ use of UCSs. Mixed results related to the use of UCSs by this population may indicate that they experience more significant barriers to help-seeking both inside and outside the university (Banties). However, as suggested by [25], if some groups of students are consistently underrepresented in services, it is unlikely that activities and interventions provided by UCSs will be appropriate for their needs, thus leading to the underutilization of these services by them.

The results of “Topic 4: Internet (web-based) delivered intervention” demonstrated significant advances in research on the effectiveness of psychological interventions, especially those regarding web-based or app-based programs (e.g., [96,100]). These findings align with the combined results of [24] and represent a huge opportunity to provide large-scale psychological support, reduce wait times, and accommodate the increasingly busy and technology-driven lifestyles of university students.

Studies included in “Topic 6: Counselors and health care providers” offered a heterogenous perspective on health care providers, since they collected results from counselors’ perspectives about counseling and clients (e.g., [32,109]) and preliminary results of effectiveness from the introduction of innovative prevention training programs with UCS staff [110]. However, the studies agreed in highlighting the need for research, training, and clinical work for counselors, which are still scarce. 

Finally, “Topic 7: Other” anticipated the forthcoming growth and development of services and interventions available from UCSs.

One of the main strengths of this systematic review is its potential to offer a broad-based look at the literature on university counseling worldwide, as opposed to the already-published review studies that focus on specific aspects of this macro topic, such as studies addressing the effectiveness of digital [23] or mobile-app based interventions [24] to improve mental health among university students, or studies estimating the use of mental health services by university students [25]. However, this also constitutes one of its main limitations, since the heterogeneity of the study designs, outcomes and measures prevented us from meta-analytically estimating the magnitude of the pooled effects, particularly regarding efficacy studies. Another limitation is the potential presence of publication bias, especially referring to studies evaluating the effectiveness of psychological interventions, that could not be empirically assessed through well-established methods (e.g., tests for funnel plot asymmetry in meta-analyses). Although this point was highlighted by previous authors [23,24], even meta-analytic studies did not assess publication bias due to the substantial between-study heterogeneity [25].

Several limitations in the existing literature must be acknowledged. Firstly, we noticed that despite increasing demand that has led to many UCSs becoming saturated [115], few studies included in our review directly measured or focused on this topic, with minimal exceptions (e.g., [36]). As a result, the existing literature provides limited support for addressing problems such as understaffing and lack of economic resources in UCSs. Moreover, studies examining counselors’ attitudes, mental health, and work satisfaction at UCSs are still lacking. Gathering their perspectives on how to improve UCSs, their performance and well-being could be a turning point to offer better services and help more students promptly and effectively. Furthermore, the diversity of both students’ needs and cultural backgrounds, as well as counselors’ psychological orientations, fields of study, culture, and expertise, may provide an extraordinary opportunity to foster counseling research and clinical training. With respect to methodological limitations, future studies should be encouraged to employ rigorous designs (e.g., blinding of treatment deliverers and outcome assessors, consideration of confounding factors) to extend our knowledge and improve the services offered, especially effectiveness studies using RCTs. Moreover, we did not collect the information on the instruments used for each study. In fact, during the screening of the articles and the data extraction, we noticed an enormous heterogeneity and diversity of instruments used. However, most of the instruments used (e.g., questionnaires, interviews) were validated tools, with good psychometric properties and DSM-oriented (especially when symptoms or well-being were assessed). Other methods were counselors’ ratings of their clients and the number of students accessing UCSs per year. Despite the heterogeneity of instruments used, we recognized extensive use of the same instrument within the experience of the Collegiate Mental Health Centre [66], namely the Counseling Center Assessment of Psychological Symptoms (CCAPS) [116], a multidimensional and psychometrically sound instrument created by counseling center staff specifically for college students and open for future refinement. Finally, since the COVID-19 outbreak represented a turning point for many mental health services, future research should document the developments of UCSs in addressing pandemic-related mental health concerns (such as [117]). In the coming years, it would be desirable to have an overview on the evaluations, care services and prevention programs implemented by UCSs for the onset and treatment of mental health disorders and psychological distress during and after the pandemic, especially in order to prevent or mitigate new crises.

## Figures and Tables

**Table 1:** Eligibility criteria.

Inclusion Criteria	Exclusion Criteria
1. English language	1. Counseling or brief intervention conducted on specific clinical populations (e.g., substance abusers)
2. Studies involving university students aged 18+ or the university community, including health care providers	2. Studies focusing on psychotherapy
3. Studies focusing on brief psychological counseling/intervention	3. Studies focusing on the COVID-19 pandemic
4. Both healthy and clinical samples	4. Studies conducted on university students without any reference to counseling services
5. All study designs were considered eligible	5. Grey literature

**Table 2:** Details regarding the types, number and sample sizes of the studies included.

Type of Study	Articles N	University Students N	Counselors N
Cross-sectional	50	232,515	2033
Longitudinal	3	2823	-
RCTs ^1^	27	7318	-
Quasi-experimental	34	76,363	78
Prevalence	10	19,329	-
Qualitative	7	291	34
Case reports	2	5	-
Text and opinion	19	-	-
Total	132	338,644	2145

^1^ Randomized control trials.

**Table 3:** List of the topics, number of included studies and geographical distribution for each topic.

TOPIC	Tot Articles *	Multi-Topics	Geographical Distribution **
1. Mental health, help-seeking behaviors and attitudes toward counseling among university students	66	17	53.73% North America, 19.40% Europe, 16.42% Asia, 4.48% Africa, 4.48% Oceania, 1.49% South America
2. Description of counseling services, good practices and purposes to improve the services	24	6	58.33% North America, 33.33% Europe, 8.33% Asia
3. Effectiveness of counseling and psychological interventions	53	21	40.74% North America, 37.04% Europe, 14.81% Asia, 7.41% Oceania
4. Internet (web-based) delivered intervention	26	26	44.44% Europe, 25.93% North America, 14.81% Oceania, 11.11% Asia, 3.70% South America
5. International students	5	5	83.33% North America, 16.67% Oceania
6. Counselors and health care providers	11	11	54.55% North America, 27.27% Asia, 9.09% Africa, 9.09% Europe
7. Other	10	8	58.33% Europe, 25.00% Asia, 8.33% Oceania, 8.33% South America

* This column includes the total number of studies addressing both the specific topic and the topic in conjunction with other topics. ** This column includes the prevalence of studies for each country per topic.

## Data Availability

Data sharing not applicable—no new data generated.

## Supplementary Materials

The following supporting information can be downloaded at: https://www.mdpi.com/article/10.3390/ejihpe13090133/s1, Document S1: Data extraction details and quality assessment; Document S2: Complete references of the articles included in the systematic review; Document S3: Detailed search strategy.
