# Supplementary material for: Psychological Counseling among University Students Worldwide: A Systematic Review
Source: Eur J Investig Health Psychol Educ. 2023 Sep 14;13(9):1831–49. doi: 10.3390/ejihpe13090133 (PMC10528000; doi:10.3390/ejihpe13090133)
Supplement: Supplementary file 1 [file ejihpe-13-00133-s001.zip › Supplementary Document S3.pdf]

Search strategy

Medline/CINAHL/PsycINFO:

1st string: psycholog* counsel* OR psycholog* intervention OR university counsel* service* OR university counsel* center* OR university counsel* centre*

AND

2nd string: university student* OR college student*

SCOPUS:

(TITLE-ABS-KEY ("psycholog* counsel*" OR "psycholog* intervention" OR "university counsel* service*" OR "university counsel* center*" OR "university counsel* centre*") AND TITLE-ABS-KEY ("university student*" OR "college student*"))

PubMed:

(psycholog* counsel*[Title/Abstract] OR psycholog* intervention[Title/Abstract] OR university counsel* service*[Title/Abstract] OR university counsel* center*[Title/Abstract] OR university counsel* centre*[Title/Abstract]) AND (university student*[Title/Abstract] OR college student*[Title/Abstract])
