# Supplementary material for: Psychological Counseling among University Students Worldwide: A Systematic Review
Source: Eur J Investig Health Psychol Educ. 2023 Sep 14;13(9):1831–49. doi: 10.3390/ejihpe13090133 (PMC10528000; doi:10.3390/ejihpe13090133)
Supplement: Supplementary file 1 [file ejihpe-13-00133-s001.zip › Supplementary Material Document S1.pdf]

Complete references of the articles included in the systematic review

1. Adamo, S.M.; Valerio, P.; Giusti, P. Psychodynamically Oriented Brief Interventions with Medical Students: An Italian Experience. *J. Coll. Stud. Psychother.* **1992**, *7*, 35–45, doi:10.1300/J035v07n02_03.
2. Adamo, S.M.G.; Fontana, M.R.; Preti, E.; Prunas, A.; Riffaldi, M.L.; Sarno, I. At the Border: Reflections on Psychoanalytically Oriented Counselling in an Italian University Setting. *Br. J. Guid. Couns.* **2012**, *40*, 5–14, doi:10.1080/03069885.2011.610437.
3. Adebowale, O.F.; Popoola, B.I. Prospects and Challenges of Online Guidance and Counselling Services in a Nigerian University. *Int. J. Adv. Couns.* **2011**, *33*, 64–78, doi:10.1007/s10447-010-9109-x.
4. Almahaireh, A.S.F.; Aldalaeen, A.S.R.; Takhaineh, S.K.A. Efficacy of a Preventive Counseling Program for Improving Psychological Hardiness and the Positive Use of Social Network Sites among Students at Risk. *Int. J. Adv. Couns.* **2018**, *40*, 173–186, doi:10.1007/s10447-018-9319-1.
5. Amodeo, A.L.; Picariello, S.; Valerio, P.; Bochicchio, V.; Scandurra, C. Group Psychodynamic Counselling with Final-Year Undergraduates in Clinical Psychology: A Clinical Methodology to Reinforce Academic Identity and Psychological Well-Being. *Psychodyn. Pract.* **2017**, *23*, 161–180, doi:10.1080/14753634.2017.1308834.
6. Ando, M. An Intervention Program Focused on Self-Understanding and Interpersonal Interactions to Prevent Psychosocial Distress among Japanese University Students. *J. Adolesc* **2011**, *34*, 929–940, doi:10.1016/j.adolescence.2010.12.003.
7. Bailey, D.; Moore, J. Closing the Distance: Counselling at Open University Residential Schools. *Br. J. Guid. Couns.* **1989**, *17*, 317–330, doi:10.1080/03069888900760291.
8. Barker, S.B.; Barker, R.T.; Schubert, C.M. Therapy Dogs on Campus: A Counseling Outreach Activity for College Students Preparing for Final Exams. *J. Coll. Couns.* **2017**, *20*, 278–288, doi:10.1002/jocc.12075.
9. Bartholomew, T. T., Gundel, B. E., Sullivan, J. W., Pérez-Rojas, A. E., Lockard, A. J. Pretreatment Counseling Experiences, Stressors, and Support Differences between Transgender and Cisgender University Students Seeking Mental Healthcare. *J. Clin. Psychol.* **2019**, *75*, 933–957, doi:10.1002/jclp.22742.
10. Bendtsen, M.; Müssener, U.; Linderoth, C.; Thomas, K. A Mobile Health Intervention for Mental Health Promotion among University Students: Randomized Controlled Trial. *JMIR Mhealth Uhealth* **2020**, *8*, doi:10.2196/17208.
11. Bernhardsdottir, J.; Vilhjalmsson, R.; Champion, J.D. Evaluation of a Brief Cognitive Behavioral Group Therapy for Psychological Distress among Female Icelandic University Students. *Issues Ment. Health Nurs.* **2013**, *34*, 497–504, doi:10.3109/01612840.2013.773473.
12. Biasi, V.; Cerutti, R.; Mallia, L.; Menozzi, F.; Patrizi, N.; Violani, C. (Mal)Adaptive Psychological Functioning of Students Utilizing University Counseling Services. *Front. Psychol.* **2017**, *8*, doi:10.3389/fpsyg.2017.00403.
13. Biasi, V.; De Vincenzo, C.; Patrizi, N.; Mosca, M.; Fagioli, S. The Combined Application of MMPI-2 and OQ-45 to Detect and Measure the Effectiveness of Psychological University Counselling. *J. Educ. Soc. Res.* **2020**, *10*, 13–23, doi:10.36941/JESR-2020-0041.
14. Biasi, V.; Patrizi, N.; Mosca, M.; De Vincenzo, C. The Effectiveness of University Counselling for Improving Academic Outcomes and Well-Being. *Br. J. Guid. Couns.* **2017**, *45*, 248–257, doi:10.1080/03069885.2016.1263826.
15. Bilodeau, C.; Meissner, J.; Antunes-Alves, S.; Konecki, R.; Ruci, L. Exploring Alliance–Outcome Associations in a Combined Mental Health and Academic Counselling Setting for At-risk Students: The Differential Role of Alliance Components, Personality, Gender and

Pre-treatment Severity. *Couns. Psychother. Res.* **2022**, *22*, 808–817, doi:10.1002/capr.12473.
16. Bird, M.D.; Chow, G.M.; Yang, Y. College Students' Attitudes, Stigma, and Intentions toward Seeking Online and Face-to-Face Counseling. *J. Clin. Psychol.* **2020**, *76*, 1775–1790, doi:10.1002/jclp.22956.
17. Bitsios, P.; Karademas, E.; Mouzaki, A.; Manolitsis, G.; Kapellaki, O.; Diacatou, A.; Archontaki, A.; Mamalakis, G.; Giovazolias, T. The Student Counselling Centre at the University of Crete, Greece. *BJPsych. Int.* **2017**, *14*, 90–92, doi:10.1192/s2056474000002099.
18. Björklund, G. Counseling at Universities in Sweden. *Int. J. Adv. Couns.* **1983**, *6*, 23–30, doi:10.1007/BF00118850.
19. Bohon, L. M., Cotter, K. A., Kravitz, R. L., Cello, P. C., Jr, Fernandez Y Garcia, E. The Theory of Planned Behavior as It Predicts Potential Intention to Seek Mental Health Services for Depression among College Students. *J. Am. Coll. Health : J of ACH* **2016**, *64*, 593–603, doi:10.1080/07448481.2016.1207646.
20. Bosmajian, C.P.; Mattson, R.E. A Controlled Study of Variables Related to Counseling Center Use. *J. Couns. Psychol.* **1980**, *27*, 510–519, doi:10.1037/0022-0167.27.5.510.
21. Bourdon, J. L., Moore, A. A., Long, E. C., Kendler, K. S., Dick, D. M. The Relationship between On-Campus Service Utilization and Common Mental Health Concerns in Undergraduate College Students. *Psychol. Serv.* **2020**, *17*, 118–126, doi:10.1037/ser0000296.
22. Bowen, N.H.; McEachern, S.K.; Pearn, P.L.; Kerr, B.A. Women Helping Women: A Peer Counseling Service. *Women Ther.* **1985**, *4*, 43–51, doi:10.1300/J015V04N02_07.
23. Boyce, R.M.; Thurlow, H.J. Characteristics of University Students with Emotional Problems. *Can. Psychiatr. Assoc. J.* **1969**, *14*, 481–492.
24. Buizza, C.; Cela, H.; Costa, A.; Ghilardi, A. Coping Strategies and Mental Health in a Sample of Students Accessing a University Counselling Service. *Couns. Psychother. Res.* **2022**, *22*, 658–666, doi:10.1002/capr.12519.
25. Buizza, C.; Ghilardi, A.; Olivetti, E.; Costa, A. Dropouts from a University Counselling Service: A Quantitative and Qualitative Study. *Br. J. Guid. Couns.* **2019**, *47*, 590–602, doi:10.1080/03069885.2019.1566513.
26. Cadaret, M.C.; Bennett, S.R. College Students' Reported Financial Stress and Its Relationship to Psychological Distress. *J. Coll. Couns.* **2019**, *22*, 225–239, doi:10.1002/jocc.12139.
27. Carney, C.G.; Savitz, C.J. Student and Faculty Perceptions of Student Needs and the Services of a University Counseling Center: Differences That Make a Difference. *J. Couns. Psychol.* **1980**, *27*, 597–604, doi:10.1037/0022-0167.27.6.597.
28. Castonguay, L.G.; Locke, B.D.; Hayes, J.A. The Center for Collegiate Mental Health: An Example of a Practice Research Network in University Counseling Centers. *J. Coll. Stud. Psychother.* **2011**, *25*, 105–119, doi:10.1080/87568225.2011.556929.
29. Celia, G.; Tessitore, F.; Cavicchiolo, E.; Girelli, L.; Limone, P.; Cozzolino, M. Improving University Students' Mental Health During the COVID-19 Pandemic: Evidence From an Online Counseling Intervention in Italy. *Front. Psychiatry* **2022**, *13*, doi:10.3389/fpsyt.2022.886538.
30. Cerutti, R.; Biuso, G.S.; Dentale, F.; Spensieri, V.; Gambardella, A.; Tambelli, R. Effectiveness of Psychodynamic-Oriented Counselling Intervention in Reducing Psychological Distress in University Students Seeking Help. *Br. J. Guid. Couns.* **2022**, doi:10.1080/03069885.2022.2089632.
31. Cerutti, R.; Fontana, A.; Ghezzi, V.; Menozzi, F.; Spensieri, V.; Tambelli, R. Exploring Psychopathological Distress in Italian University Students Seeking Help: A Picture from a

University Counselling Service. *Curr. Psychol.* **2022**, *41*, 1382–1394, doi:10.1007/s12144-020-00665-9.
32. Ceyhan, E.; Ceyhan, A.A. Loneliness and Depression Levels of Students Using a University Counseling Center. *Egit. ve Bilim* **2011**, *36*, 81–92.
33. Chang, T.; Chang, R. Counseling and the Internet: Asian American and Asian International College Students' Attitudes Toward Seeking Online Professional Psychological Help. *J. Coll. Couns.* **2004**, *7*, 140–149, doi:10.1002/j.2161-1882.2004.tb00245.x.
34. Chugani, C.D. Adapting Dialectical Behavior Therapy for College Counseling Centers. *J. Coll. Couns.* **2017**, *20*, 67–80, doi:10.1002/jocc.12059.
35. D'Amico, N., Mechling, B., Kemppainen, J., Ahern, N. R., Lee, J. American College Students' Views of Depression and Utilization of On-Campus Counseling Services. *J. Am. Psychiatr. Nurses Assoc.* **2016**, *22*, 302–311, doi:10.1177/1078390316648777.
36. De Fabritiis, M.; Trisolini, F.; Bertuletti, G.; Fagadau, I.D.; Ginelli, D.; Lalopa, K.P.; Peverelli, L.; Pirola, A.; Sala, G.; Maisto, M.; et al. An Internet-Based Multi-Approach Intervention Targeting University Students Suffering from Psychological Problems: Design, Implementation, and Evaluation. *Int. J. Environ. Res. Public Health* **2022**, *19*, doi:10.3390/ijerph19052711.
37. de Oliveira, M.L.C.; de Rosalmeida Dantas, C.; de Azevedo, R.C.S.; Banzato, C.E.M. Counseling Brazilian Undergraduate Students: 17 Years of a Campus Mental Health Service. *Int. J. Environ. Res. Public Health* **2008**, *57*, 367–372, doi:10.3200/JACH.57.3.367-372.
38. de Vries, S.R.; Valadez, A.A. Counseling Students' Mental Health Status and Attitudes toward Counseling. *J. Prof. Couns.* **2006**, *34*, 77–87.
39. Deane, F.P.; Todd, D.M. Attitudes and Intentions to Seek Professional Psychological Help for Personal Problems or Suicidal Thinking. *J. Coll. Stud. Psychother.* **1996**, *10*, 45–59, doi:10.1300/J035v10n04_06.
40. Dederichs, M.; Weber, J.; Muth, T.; Angerer, P.; Loerbroks, A. Students' Perspectives on Interventions to Reduce Stress in Medical School: A Qualitative Study. *PLoS ONE* **2020**, *15*, doi:10.1371/journal.pone.0240587.
41. Der Pan, P. J., Fan, A. C., Bhat, C. S., Chang, S. S. H. Associations among Self-Concept, Verbal Behaviors, and Group Climate Early in the Group Counseling Process. *Psychol. Rep.* **2012**, *111*, 739–754, doi:10.2466/02.17.21.PR0.111.6.739-754.
42. Di Consiglio, M., Merola, S., Satta, C., Pascucci, T., Violani, C., Couyoumdjian, A. NoiBene, a Group Intervention for Promoting Mental Health Among University Students: A Study Protocol for a Randomized Controlled Trial. *Front. Psychol.* **2022**, *13*, 877340, doi:10.3389/fpsyg.2022.877340.
43. Di Consiglio, M.; Fabrizi, G.; Conversi, D.; La Torre, G.; Pascucci, T.; Lombardo, C.; Violani, C.; Couyoumdjian, A. Effectiveness of NoiBene: A Web-Based Programme to Promote Psychological Well-Being and Prevent Psychological Distress in University Students. *Appl. Psychol. Health Well-Being* **2021**, *13*, 317–340, doi:10.1111/aphw.12251.
44. Dogan, T. Problem Areas of Students at a University Psychological Counselling Centre: A 16-Year Analysis. *Br. J. Guid. Couns.* **2018**, *46*, 429–440, doi:10.1080/03069885.2018.1437255.
45. Dreman, S.B. Expectations and Preferences of Clients for a University Student Counseling Service. *J. Couns. Psychol.* **1977**, *24*, 459–462, doi:10.1037/0022-0167.24.5.459.
46. Ebert, D. D., Franke, M., Kählke, F., Küchler, A. M., Bruffaerts, R., Mortier, P., ... WHO World Mental Health–International College Student collaborators. Increasing intentions to use mental health services among university students. Results of a pilot randomized controlled trial within the World Health Organization's World Mental Health International College Student Initiative. *Int. J. Methods Psychiatr. Res.* **2019,** *28*(2), e1754. doi:10.1002/mpr.1754.

47. Epton, T., Norman, P., Dadzie, A. S., Harris, P. R., Webb, T. L., Sheeran, P., Julious, S. A., Ciravegna, F., Brennan, A., Meier, P. S., Naughton, D., Petroczi, A., Kruger, J., Shah, I. A Theory-Based Online Health Behaviour Intervention for New University Students (U@Uni): Results from a Randomised Controlled Trial. *BMC public health* **2014**, *14*, 563, doi:10.1186/1471-2458-14-563.
48. Erdem, A.; Bardakci, S.; Erdem, Ş. Receiving Online Psychological Counseling and Its Causes: A Structural Equation Model. *Curr. Psychol.* **2018**, *37*, 591–601, doi:10.1007/s12144-016-9542-z.
49. Erdur-Baker, O.; Aberson, C.L.; Barrow, J.C.; Draper, M.R. Nature and Severity of College Students' Psychological Concerns: A Comparison of Clinical and Nonclinical National Samples. *Prof. Psychol.: Res. Pract.* **2006**, *37*, 317–323, doi:10.1037/0735-7028.37.3.317.
50. Eritsyan, K.; Antonova, N.; Kazantseva, T.; Usacheva, N. Association between Descriptive and Injunctive Norms and Students' Intentions to Seek Support from Mental Health Services. *Couns. Psychother. Res.* **2021**, *21*, 827–836, doi:10.1002/capr.12461.
51. Esposito, G.; Passeggia, R.; Pepicelli, G.; Cannata, A.; Parlato, F.; Freda, M.F. Mentalizing the University Experience: An Exploratory Study on the Relationship between University Students' Reflective Functioning, Psychological Well-Being and Academic Performance. *Mediterr. J. Clin. Psychol.* **2020**, *8*, 1–21, doi:10.6092/2282-1619/mjcp-2415.
52. Fandie, K.; Naudé, L. 'Being Flawed or Being Courageous': South African Students' Attitudes towards Counseling. *Curr. Psychol.* **2019**, *38*, 647–658, doi:10.1007/s12144-017-9645-1.
53. Fang, L.; Tarshis, S.; McInroy, L.; Mishna, F. Undergraduate Student Experiences with Text-Based Online Counselling. *Br. J. Soc. Work* **2018**, *48*, 1774–1790, doi:10.1093/bjsw/bcx111.
54. Frazier, P., Meredith, L., Greer, C., Paulsen, J. A., Howard, K., Dietz, L. R., Qin, K. Randomized Controlled Trial Evaluating the Effectiveness of a Web-Based Stress Management Program among Community College Students. *Anxiety Stress Coping* **2015**, *28*, 576–586, doi:10.1080/10615806.2014.987666.
55. Fuchs, D.C.; Eskridge, E.R.; Sacks, D.N.; Porter, M.; Parks-Piatt, J. Multidisciplinary Treatment Planning: An Educational and Administrative Tool for Resource Management in a University Counseling Center. *Acad. Psychiatry* **2015**, *39*, 541–543, doi:10.1007/s40596-014-0262-0.
56. Giddan, N.S.; Levy, D.M.; Estroff, R.M.; Cline, J.C.; Altman, E.; Isham, K.; Weiss, S. College Counseling and Student Retention: Data and Speculations. *J. Coll. Stud. Psychother.* **1987**, *1*, 5–28, doi:10.1300/J035v01n03_02.
57. Giovazolias, T.; Leontopoulou, S.; Triliva, S. Assessment of Greek University Students' Counselling Needs and Attitudes: An Exploratory Study. *Int. J. Adv. Couns.* **2010**, *32*, 101–116, doi:10.1007/s10447-010-9092-2.
58. Givens, J.L.; Tjia, J. Depressed Medical Students' Use of Mental Health Services and Barriers to Use. *Acad. Med.* **2002**, *77*, 918–921, doi:10.1097/00001888-200209000-00024.
59. Gloria, A.M.; Rodriguez, E.R. Counseling Latino University Students: Psychosociocultural Issues for Consideration. *J. Couns. Dev.* **2000**, *78*, 145–154, doi:10.1002/j.1556-6676.2000.tb02572.x.
60. Green, J.L.; Lowry, J.L.; Kopta, S.M. College Students versus College Counseling Center Clients: What Are the Differences? *J. Coll. Stud. Psychother.* **2003**, *17*, 25–37, doi:10.1300/J035v17n04_05.
61. Grégoire, S.; Lachance, L.; Bouffard, T.; Dionne, F. The Use of Acceptance and Commitment Therapy to Promote Mental Health and School Engagement in University Students: A Multisite Randomized Controlled Trial. *Behav. Ther.* **2018**, *49*, 360–372, doi:10.1016/j.beth.2017.10.003.

62. Güneri, O.Y.; Aydın, G.; Skovholt, T. Counseling Needs of Students and Evaluation of Counseling Services at a Large Urban University in Turkey. *Int. J. Adv. Couns.* **2003**, *25*, 53–63, doi:10.1023/A:1024928212103.
63. Haeger, J.A.; Davis, C.H.; Levin, M.E. Utilizing ACT Daily as a Self-Guided App for Clients Waiting for Services at a College Counseling Center: A Pilot Study. *J. Am. Coll. Health.* **2022**, *70*, 742–749, doi:10.1080/07448481.2020.1763366.
64. Hardy, J.A.; Weatherford, R.D.; Locke, B.D.; Depalma, N.H.; D'Iuso, N.T. Meeting the Demand for College Student Concerns in College Counseling Centers: Evaluating a Clinical Triage System. *J. Coll. Stud. Psychother.* **2011**, *25*, 220–240, doi:10.1080/87568225.2011.581929.
65. Harrington, K.D.; Eres, R.; Lim, M.H. The Web-Based Uprise Program for Mental Health in Australian University Students: Protocol for a Pilot Randomized Controlled Trial. *JMIR Res. Protoc.* **2020**, *9*, doi:10.2196/21307.
66. Herrero, R., Mira, A., Cormo, G., Etchemendy, E., Baños, R., García-Palacios, A., Ebert, D. D., Franke, M., Berger, T., Schaub, M. P., Görlich, D., Jacobi, C., Botella, C. An Internet Based Intervention for Improving Resilience and Coping Strategies in University Students: Study Protocol for a Randomized Controlled Trial. *Internet interv.* **2019**, *16*, 43–51, doi:10.1016/j.invent.2018.03.005.
67. House, L.A.; Neal, C.; Kolb, J. Supporting the Mental Health Needs of First Generation College Students. *J. Coll. Stud. Psychother.* **2020**, *34*, 157–167, doi:10.1080/87568225.2019.1578940.
68. Houston, J. B., First, J., Spialek, M. L., Sorenson, M. E., Mills-Sandoval, T., Lockett, M., First, N. L., Nitiéma, P., Allen, S. F., Pfefferbaum, B. Randomized Controlled Trial of the Resilience and Coping Intervention (RCI) with Undergraduate University Students. *J. Am. Coll. Health.* **2017**, *65*, 1–9, doi:10.1080/07448481.2016.1227826.
69. Hwang, B.J.; Bennett, R.; Beauchemin, J. International Students' Utilization of Counseling Services. *Coll. Stud. J.* **2014**, *48*, 347–354.
70. Hyun, J.K.; Quinn, B.C.; Madon, T.; Lustig, S. Graduate Student Mental Health: Needs Assessment and Utilization of Counseling Services. *J. Coll. Stud. Dev.* **2006**, *47*, 247–266, doi:10.1353/csd.2006.0030.
71. Ikonomopoulos, J.; Garza, K.; Weiss, R.; Morales, A. Examination of Treatment Progress Among College Students in a University Counseling Program. *Couns. Outcome Res. Evaluation* **2021**, *12*, 30–42, doi:10.1080/21501378.2020.1850175.
72. Jankauskaite, G.; O'Brien, K.M.; Yang, N. Assessing Knowledge and Predicting Grief Counseling Skills Among University Counseling Center Therapists. *Couns. Psychol.* **2021**, *49*, 458–484, doi:10.1177/0011000020983525.
73. Karyotaki, E.; Klein, A. M.; Riper, H.; De Wit, L.; Krijnen, L.; Bol, E.; ... & Cuijpers, P. Examining the effectiveness of a web-based intervention for symptoms of depression and anxiety in college students: study protocol of a randomised controlled trial. *Bmj Open* **2019**, *9*(5), e028739. doi: 10.1136/bmjopen-2018-028739
74. Kaufman, J.A. Stress and Social Support Among Online Doctoral Psychology Students. *J. Coll. Stud. Psychother.* **2006**, *20*, 79–88, doi:10.1300/J035v20n03_07.
75. Kearney, L. K., Draper, M., Barón, A. Counseling Utilization by Ethnic Minority College Students. *Cultur. Divers. Ethnic Minor. Psychol.* **2005**, *11*, 272–285, doi:10.1037/1099-9809.11.3.272.
76. Kemp, A.D. Counseling Center Psychologists in Neuropsychology: Counseling Neuropsychology. *The Counseling Psychologist* **1992**, *20*, 571–604, doi:10.1177/0011000092204003.
77. Kim, J.E.; Park, S.S.; La, A.; Chang, J.; Zane, N. Counseling Services for Asian, Latino/a, and White American Students: Initial Severity, Session Attendance, and Outcome. *Cultural Diversity and Ethnic Minority Psychology* **2016**, *22*, 299–310, doi:10.1037/cdp0000069.

78. Kivlighan, I., D.M.; Schreier, B.A.; Gates, C.; Hong, J.E.; Corkery, J.M.; Anderson, C.L.; Keeton, P.M. The Role of Mental Health Counseling in College Students' Academic Success: An Interrupted Time Series Analysis. *Journal of Counseling Psychology* **2020**, doi:10.1037/cou0000534.
79. Kounenou, K., Kalamatianos, A., Garipi, A., Kourmousi, N. Positive Psychology Group Intervention in Greek University Students by the Counseling Center: Effectiveness of Implementation. *Front. Psychol.* **2022**, *13*, 965945, doi:10.3389/fpsyg.2022.965945.
80. Koutra, A.; Katsiadrami, A.; Diakogiannis, G. The Effect of Group Psychological Counselling in Greek University Students' Anxiety, Depression, and Self-Esteem. *European Journal of Psychotherapy and Counselling* **2010**, *12*, 101–111, doi:10.1080/13642537.2010.482733.
81. Koydemir, S.; Sun-Selışık, Z.E. Well-Being on Campus: Testing the Effectiveness of an Online Strengths-Based Intervention for First Year College Students. *British Journal of Guidance & Counselling* **2016**, *44*, 434–446, doi:10.1080/03069885.2015.1110562.
82. Lanman, M. Psychoanalytic Psychotherapy and Student Counselling. *Psychoanalytic Psychotherapy* **1994**, *8*, 129–140, doi:10.1080/02668739400700141.
83. Lattie, E. G., Cohen, K. A., Hersch, E., Williams, K. D. A., Kruzan, K. P., MacIver, C., Hermes, J., Maddi, K., Kwasny, M., & Mohr, D. C. Uptake and Effectiveness of a Self-Guided Mobile App Platform for College Student Mental Health. *Internet Interv.* **2022**, *27*, 100493, doi:10.1016/j.invent.2021.100493.
84. Lin, A.P.C.; Trappey, C.V.; Luan, C.-C.; Trappey, A.J.C.; Tu, K.L.K. A Test Platform for Managing School Stress Using a Virtual Reality Group Chatbot Counseling System. *Appl. Sci.s (Switzerland)* **2021**, *11*, doi:10.3390/app11199071.
85. Lockard, A.J.; Hayes, J.A.; McAleavey, A.A.; Locke, B.D. Change in Academic Distress: Examining Differences between a Clinical and Nonclinical Sample of College Students. *J. Coll. Couns.* **2012**, *15*, 233–246, doi:10.1002/j.2161-1882.2012.00018.x.
86. Loeffler, D.; Fiedler, L. Woman—A Sense of Identity: A Counseling Intervention to Facilitate Personal Growth in Women. *J. Couns. Psychol* **1979**, *26*, 51–57, doi:10.1037/0022-0167.26.1.51.
87. Lucas, M.S. A Validation of Types of Career Indecision at a Counseling Center. *J. Couns. Psychol.***1993**, *40*, 440–446, doi:10.1037/0022-0167.40.4.440.
88. Mahon, M.; Laux, J.M.; McGuire Wise, S.; Ritchie, M.H.; Piazza, N.J.; Tiamiyu, M.F. Brief Therapy at a University Counseling Center: Working Alliance, Readiness to Change, and Symptom Severity. *J. Coll. Couns.* **2015**, *18*, 233–243, doi:10.1002/jocc.12017.
89. Mallinckrodt, B.; Shigeoka, S.; Suzuki, L.A. Asian and Pacific Island American Students' Acculturation and Etiology Beliefs about Typical Counseling Presenting Problems. *Cult. Divers. Ethn. Minor. Psychol.* **2005**, *11*, 227–238, doi:10.1037/1099-9809.11.3.227.
90. Markin, R.D.; McCarthy, K.S.; Hayes, J.A. Young Pregnant Clients in College or University Counselling Centres: Environmental and Symptom Experiences. *Couns. Psychother. Res.* **2021**, *21*, 768–780, doi:10.1002/capr.12445.
91. McCormick, R.M.; Paterson, D.W. Student Counselling in Canadian Universities. *Int. J. Adv. Couns.* **1996**, *18*, 235–243, doi:10.1007/BF01408098.
92. McKenzie, K.; Murray, K.R.; Murray, A.L.; Richelieu, M. The Effectiveness of University Counselling for Students with Academic Issues. *Couns. Psychother. Res* **2015**, *15*, 284–288.
93. McLennan, M.W.; Sutton, R.H. Stress in Veterinary Science Students: A Study at the University of Queensland. *Vet Med Educ***2005**, *32*, 213–218, doi:10.3138/jvme.32.2.213.
94. McLeod, M.; Tercek, T.; Wisbey, M. Facilitating Mental Health on College Campuses: Consultation between University Counseling Centers and Offices of Residence Life. *J. Am. Coll. Health* **1985**, *33*, 168–170, doi:10.1080/07448481.1985.9936183.

95. Menozzi, F.; Gizzi, N.; Tucci, M.T.; Patrizi, N.; Mosca, M. Emotional Dysregulation: The Clinical Intervention of Psychodynamic University Counselling. *J. Educ. Cult. Psychol. Stud.***2016**, *2016*, 169–182, doi:10.7358/ecps-2016-014-meno.
96. Morse, C.C.; Spoltore, J.D.; Galvinhill, P. College/University Counseling Centers Supporting Study Away: Challenges and Opportunities. *J. Coll. Stud. Psychother.***2017**, *31*, 325–335, doi:10.1080/87568225.2017.1313690.
97. Murray, A.L.; McKenzie, K.; Murray, K.R.; Richelieu, M. An Analysis of the Effectiveness of University Counselling Services. *Br. J. Guid. Couns.***2016**, *44*, 130–139, doi:10.1080/03069885.2015.1043621.
98. Musiat, P.; Conrod, P.; Treasure, J.; Tylee, A.; Williams, C.; Schmidt, U Targeted Prevention of Common Mental Health Disorders in University Students: Randomised Controlled Trial of a Transdiagnostic Trait-Focused Web-Based Intervention. *PloS one* **2014**, *9*, e93621, doi:10.1371/journal.pone.0093621.
99. Musiat, P.; Potterton, R.; Gordon, G.; Spencer, L.; Zeiler, M.; Waldherr, K.; ... & Schmidt, U. Web-Based Indicated Prevention of Common Mental Disorders in University Students in Four European Countries - Study Protocol for a Randomised Controlled Trial. *Internet interv*. **2019**, *16*, 35–42, doi:10.1016/j.invent.2018.02.004.
100. Musso, P.; Coppola, G.; Pantaleo, E.; Amoroso, N.; Balenzano, C.; Bellotti, R.; Cassibba, R.; Diacono, D.; Monaco, A. Psychological Counseling in the Italian Academic Context: Expected Needs, Activities, and Target Population in a Large Sample of Students. *PLoS ONE* **2022**, *17*, doi:10.1371/journal.pone.0266895.
101. Nam, S. K., Choi, S. I., Lee, S. M. Effects of Stigma-Reducing Conditions on Intention to Seek Psychological Help among Korean College Students with Anxious-Ambivalent Attachment. *Psychol. Serv.* **2015**, *12*, 167–176, doi:10.1037/a0038713.
102. Negash, A., Khan, M. A., Medhin, G., Wondimagegn, D., Araya, M. Mental distress, perceived need, and barriers to receive professional mental health care among university students in Ethiopia. *BMC Psychiatry* **2020**, *20*(1), 1-15.
103. Newman, M.L.; Greenway, P. Therapeutic Effects of Providing MMPI-2 Test Feedback to Clients at a University Counseling Service: A Collaborative Approach. *Psychol. Assess.* **1997**, *9*, 122–131, doi:10.1037/1040-3590.9.2.122.
104. Niileksela, C. R., Ghosh, A., Janis, R. A. Dynamic Changes in Generalized Anxiety and Depression during Counseling. *J. Couns. Psychol.* **2021**, *68*, 112–124, doi:10.1037/cou0000441.
105. Ning, X., Wong, J. P., Huang, S., Fu, Y., Gong, X., Zhang, L., Hilario, C., Fung, K. P., Yu, M., Poon, M. K., Cheng, S., Gao, J., Jia, C. X. Chinese University Students' Perspectives on Help-Seeking and Mental Health Counseling. *Int. J. Environ. Res. Public Health* **2022**, *19*, doi:10.3390/ijerph19148259.
106. Nozawa, H.; Ikegami, K.; Michii, S.; Sugano, R.; Ando, H.; Kitamura, H.; Ogami, A. Peer Counseling for Mental Health in Young People—Randomized Clinical Trial –. *Ment. Health Prev* **2019**, *14*, doi:10.1016/j.mph.2019.200164.
107. O'Shea, A.,Kilcullen, J..R., Hayes, J., Scofield, B. Examining the effectiveness of campus counseling for college students with disabilities. *Rehabil. Psychol.* **2021**, *66*(3), 300–310. https://doi.org/10.1037/rep0000349
108. Pace, C.S.; Usai, M.C.; Bizzi, F.; Minetto, P.; Alcetti, A.; Zanobini, M. Psychological Counseling Service "Together" at University of Genoa: Students' Psychological Profile in Pre and Post Pandemic. *Front. Psychol* **2022**, *13*, doi:10.3389/fpsyg.2022.898530.
109. Palma-Gòmez, R, Herrero., R, Banos, R., Garcìa-Palacios, A.,Castaneiras, C., Fernandez, G.L., Llull, D.M., Torres, L.C., Barraco, L.A., Càrdenas-Gòmez, L., Botella, C. Efficacy of a self-applied online program to promote resilience and coping skills in university students in four Spanish-speaking countries: Study protocol for a randomized

controlled trial. *BMC Psychiatry* **2020**, *20*(1), 148. https://doi.org/10.1186/s12888-020-02536-w
110. Patrzek, J.; Grunschel, C.; Fries, S. Academic Procrastination: The Perspective of University Counsellors. *Int. J. Adv. Couns* **2012**, *34*, 185–201, doi:10.1007/s10447-012-9150-z.
111. Pérez-Rojas, A. E., Lockard, A. J., Bartholomew, T. T., Janis, R. A., Carney, D. M., Xiao, H., Youn, S. J., Scofield, B. E., Locke, B. D., Castonguay, L. G., Hayes, J. A. Presenting concerns in counseling centers: The view from clinicians on the ground. *Psychol. Serv.* **2017**, *14*(4), 416–427. https://doi.org/10.1037/ser0000122
112. Peykari, N.; Tehrani, F.R.; Afzali, H.M.; Eftekhari, M.B.; Djalalinia, S. The Key Stakeholders' Opinions Regarding University Counseling Centers: An Experience from Iran. *J. Res. Med. Sci.* **2011**, *16*, 1202–1209.
113. Pinkerton, R.S.; Kenneth Rockwell, W.J. Very Brief Psychological Interventions with University Students. *J. Am. Coll. Health* **1994**, *42*, 156–162, doi:10.1080/07448481.1994.9939663.
114. Ponterotto, J.G.; Rao, V.; Zweig, J.; Rieger, B.P.; Schaefer, K.; Michelakou, S.; Armenia, C.; Goldstein, H. The Relationship of Acculturation and Gender to Attitudes toward Counseling in Italian and Greek American College Students. *Cult. Divers. Ethn. Minor. Psychol.* **2001**, *7*, 362–375, doi:10.1037/1099-9809.7.4.362.
115. Prasath, P.R.; Lim, A.L.S.; Steen, S. A Strength-Based Support Group for International College Students: A Pilot Study. *J. Spec. Group Work* **2022**, *47*, 63–82, doi:10.1080/01933922.2021.2000085.
116. Räsänen, P.; Lappalainen, P.; Muotka, J.; Tolvanen, A.; Lappalainen, R. An Online Guided ACT Intervention for Enhancing the Psychological Wellbeing of University Students: A Randomized Controlled Clinical Trial. *Behav. Res. Ther.* **2016**, *78*, 30–42, doi:10.1016/j.brat.2016.01.001.
117. Rith-Najarian, L.; Sun, W.; Chen, A.; Chorpita, B.; Chavira, D.; Mougalian, S.; Gong-Guy, E. What's in a Name? Branding of Online Mental Health Programming for University Students. *J. Consult. Clin. Psychol*. **2019**, *87*, 380–391, doi:10.1037/ccp0000383.
118. Riva Crugnola, C.; Bottini, M.; Madeddu, F.; Preti, E.; Ierardi, E. Psychological Distress and Attachment Styles in Emerging Adult Students Attending and Not Attending a University Counselling Service. *Health Psychol. Open* **2021**, *8*, doi:10.1177/20551029211016120.
119. Saleh, D., Camart, N., Sbeira, F., Romo, L. Can We Learn to Manage Stress? A Randomized Controlled Trial Carried out on University Students. *PloS one* **2018**, *13*, e0200997, doi:10.1371/journal.pone.0200997.
120. Salisbury, H. Counseling Center Name and Type of Problems Referred. *J. Couns. Psychol.* **1972**, *19*, 351–352, doi:10.1037/h0033082.
121. Sapadin, K., Hollander, B. L. Distinguishing the Need for Crisis Mental Health Services among College Students. *Psychol. Serv.* **2022**, *19*, 317–326, doi:10.1037/ser0000526.
122. Savarese, G.; Iannaccone, A.; Mollo, M.; Fasano, O.; Pecoraro, N.; Carpinelli, L.; Cavallo, P. Academic Performance-Related Stress Levels and Reflective Awareness: The Role of the Elicitation Approach in an Italian University's Psychological Counselling. *Br. J. Guid. Couns.* **2019**, *47*, 569–578, doi:10.1080/03069885.2019.1600188.
123. Schmidt, L.; Chock, S. Counseling Psychology at Ohio State University: The First 50 Years. *J. Couns. Dev.* **1990**, *68*, 276–281, doi:10.1002/j.1556-6676.1990.tb01373.x.
124. Schneidler, G.G.; Berdie, R.F. Representativeness of College Students Who Receive Counseling Services. *J. Educ. Psychol.* **1942**, *33*, 545–551, doi:10.1037/h0057372.
125. Seppälä, E. M., Bradley, C., Moeller, J., Harouni, L., Nandamudi, D., & Brackett, M. A. Promoting Mental Health and Psychological Thriving in University Students: A

Randomized Controlled Trial of Three Well-Being Interventions. *Front. Psychiatry* **2020**, *11*, 590, doi:10.3389/fpsyt.2020.00590.
126. Sharf, R.S.; Bishop, J.B. Adjustment Differences between Counseled and Noncounseled Students at a University Counseling Center. *J. Couns. Psychol*. **1973**, *20*, 509–512, doi:10.1037/h0035191.
127. Sharif, F.; Armitage, P. The Effect of Psychological and Educational Counselling in Reducing Anxiety in Nursing Students. *J. Psychiatr. Ment. Health Nurs.* **2004**, *11*, 386–392, doi:10.1111/j.1365-2850.2003.00720.x.
128. Snider, P.D. Counselling for International Students in Western Universities: A Cross-Cultural Examination of Counselling Expectations and Services. *Int. Educ. J.* **2001**, *2*, 61–85.
129. Snyder, J.F.; Hill, C.E.; Derksen, T.P. Why Some Students Do Not Use University Counseling Facilities. *J. Couns. Psychol*. **1972**, *19*, 263–268, doi:10.1037/h0033075.
130. Solberg, V.S.; Ritsma, S.; Davis, B.J.; Tata, S.P.; Jolly, A. Asian-American Students' Severity of Problems and Willingness to Seek Help From University Counseling Centers: Role of Previous Counseling Experience, Gender, and Ethnicity. *J. Couns. Psychol.* **1994**, *41*, 275–279, doi:10.1037/0022-0167.41.3.275.
131. Stallman, H.M.; Ohan, J.L.; Chiera, B. Reducing Distress in University Students: A Randomised Control Trial of Two Online Interventions. *Aust. Psychol.* **2019**, *54*, 125–131, doi:10.1111/ap.12375.
132. Stewart, K. L., Darling, E. V., Yen, S., Stanley, B., Brown, G. K., Weinstock, L. M. Dissemination of the Safety Planning Intervention (SPI) to University Counseling Center Clinicians to Reduce Suicide Risk Among College Students. *Arch. Suicide Res.* **2020**, *24*, 75–85, doi:10.1080/13811118.2018.1531797.
133. Stewart, S.M.; Betson, C.; Marshall, I.; Wong, C.M.; Lee, P.W.; Lam, T.H. Stress and Vulnerability in Medical Students. *Med. Educ.***1995**, *29*, 119–127, doi:10.1111/j.1365-2923.1995.tb02814.x.
134. Strepparava, M.G.; Bani, M.; Zorzi, F.; Corrias, D.; Dolce, R.; Rezzonico, G. Cognitive Counselling Intervention: Treatment Effectiveness in an Italian University Centre. *Br. J. Guid. Couns.* **2016**, *44*, 423–433, doi:10.1080/03069885.2015.1110561.
135. Strepparava, M.G.; Bani, M.; Zorzi, F.; Mazza, U.; Barile, F.; Rezzonico, G. Does the Severity of Psychopathology of Italian Students Receiving Counselling Services Increase over Time? A 5-Year Analysis and a Comparison with a Clinical and Non-Clinical Sample. *Clin. Psychol. Psychother* **2017**, *24*, O1448–O1454, doi:10.1002/cpp.2096.
136. Sue, D.W.; Kirk, B.A. Asian-Americans: Use of Counseling and Psychiatric Services on a College Campus. *J. Couns. Psychol.* **1975**, *22*, 84–86, doi:10.1037/h0076153.
137. Surtees, P.G.; Pharoah, P.D.P.; Wainwright, N.W.J. A Follow-up Study of New Users of a University Counselling Service. *Br. J. Guid. Couns.* **1998**, *26*, 255–272, doi:10.1080/03069889800760221.
138. Tedeschi, G.J.; Willis, F.N. Attitudes toward Counseling among Asian International and Native Caucasian Students. *J. Coll. Stud. Psychother.* **1993**, *7*, 43–54, doi:10.1300/J035v07n04_04.
139. Thuryrajah, V.; Ahmed, E.M.; Nathan, R.J. Do Public Universities Have More Effective Counselling Services than Private Universities in Melaka? *Couns. Psychother. Res.* **2020**, *20*, 300–308, doi:10.1002/capr.12265.
140. Tidwell, R.; Hanassab, S. New Challenges for Professional Counsellors: The Higher Education International Student Population. *Couns. Psychol. Q.* **2007**, *20*, 313–324, doi:10.1080/09515070701573927.
141. Turner, A.L.; Berry, T.R. Counseling Center Contributions to Student Retention and Graduation: A Longitudinal Assessment. *J. Coll. Stud. Dev.* **2000**, *41*, 627–636.

142. Viskovich, S., Pakenham, K. I. Pilot Evaluation of a Web-Based Acceptance and Commitment Therapy Program to Promote Mental Health Skills in University Students. *J. Clin. Psychol.* **2018**, *74*, 2047–2069, doi:10.1002/jclp.22656.
143. Viskovich, S., Pakenham, K. I.. Randomized Controlled Trial of a Web-Based Acceptance and Commitment Therapy (ACT) Program to Promote Mental Health in University Students. *J. Clin. Psychol.* **2020**, *76*, 929–951, doi:10.1002/jclp.22848.
144. Vonk, M.E.; Thyer, B.A. Evaluating the Effectiveness of Short-Term Treatment at a University Counseling Center. *J. Clin. Psychol.* **1999**, *55*, 1095–1106, doi:10.1002/(sici)1097-4679(199909)55:9<1095::aid-jclp7>3.0.co;2-a.
145. Watanabe-Muraoka, A.M. The National System of University Counselling in Japan. *Int. J. Adv. Couns.* **1996**, *19*, 15–27, doi:10.1007/BF00121004.
146. Watts, J.R.; Chumbler, N.R.; Sharma, R.; Baniya, G. Examining the Mental Health Needs of Post-Secondary Students with Child-Maltreatment Histories Seeking Campus Counseling Services. *J. Coll. Stud. Psychother.* **2022**, doi:10.1080/87568225.2022.2098001.
147. Williamson, E.G.; Longstaff, H.P.; Edmonds, J.M. Counseling Arts College Students. *J. Appl. Psychol.* **1935**, *19*, 111–124, doi:10.1037/h0054251.
148. Yang, H.; Guan, Y. The Effect of Positive Rumination Training on Mental Health and Attentional Bias. *J. Psychopathol. Behav. Ass.* **2022**, *44*, 582–589, doi:10.1007/s10862-022-09959-7.
149. Yang, Q.; Shi, Q.; Zhang, L. Mental Health Problems and Counseling Path for College Students Preparing for Exams. *Rev. Argentina de Clin. Psicol.* **2020**, *29*, 1364–1369, doi:10.24205/03276716.2020.197.
150. Zaman, R.M. Psychological Problems of Medical Students in Pakistan: Data from the Aga Khan University, Karachi. *Teach. Learn. Med.* **1996**, *8*, 19–22, doi:10.1080/10401339609539758.
151. Zamostny, K.P.; Corrigan, J.D.; Eggert, M.A. Replication and Extension of Social Influence Processes in Counseling: A Field Study. *J. Couns. Psychol.* **1981**, *28*, 481–489, doi:10.1037/0022-0167.28.6.481.
152. Zarski, J.J.; Sweeney, T.J.; Barcikowski, R.S. Counseling Effectiveness as a Function of Counselor Social Interest. *J. Couns. Psychol.* **1977**, *24*, 1–5, doi:10.1037/0022-0167.24.1.1.
